# Concomitant acute pulmonary embolism, myocardial infarction and ischemic stroke due to paradoxical embolism from a patent foramen ovale: a case report

**DOI:** 10.1093/omcr/omab101

**Published:** 2021-10-26

**Authors:** Kiyoshi Takemoto, Michitaka Nakamura, Kazuaki Atagi

**Affiliations:** Division of Critical Care Medicine, Nara Prefecture General Medical Center, Nara City, Nara, 6308581, Japan

## Abstract

A patent foramen ovale (PFO) is a cause of paradoxical embolism. Although most patients with a PFO are asymptomatic, various clinical manifestations may be associated with PFO. The most important is a cryptogenic stroke. Concomitant acute pulmonary embolism (APE), acute myocardial infarction (AMI) and acute ischemic stroke (AIS) due to paradoxical embolism from a PFO are extremely rare. We describe a 77-year-old woman with a past medical history of hypertension who was transferred due to a sudden onset of dyspnea followed by cardiopulmonary arrest. Based on the patient’s medical history, transthoracic and transesophageal echocardiography, coronary angiography, and a whole-body contrasted computed tomography, we diagnosed concomitant APE, AMI and AIS caused by a paradoxical embolism from a PFO. Appropriate knowledge of the pathophysiology of this rare critical illness is important for prompt diagnosis and treatment.

## INTRODUCTION

A patent foramen ovale (PFO) occurs in ~20–30% of the general population and is a major cause of paradoxical embolism [1]. A possible mechanism for PFO-related systemic embolic events is a paradoxical embolism, a thrombus from the peripheral venous system that crosses an interatrial defect into the systemic circulation [2]. It is rare to find acute myocardial infarction (AMI) due to paradoxical embolism [3], and its incidence has been reported as <1% [4]. Although several AMI cases studies alone or with concomitant AMI and acute pulmonary embolism (APE) with paradoxical embolism due to a PFO have been reported [5–7], concomitant AMI, APE and acute ischemic stroke (AIS) resulting in cardiac arrest are extremely rare. We describe a case of a patient with paradoxical embolism as concomitant APE, AMI and AIS. The patient’s medical history suggested that APE caused cardiac arrest, and pulmonary hypertension from APE led to a right-to-left shunt due to the PFO, which resulted in AMI and AIS.

## CASE PRESENTATION

**Figure 1:**
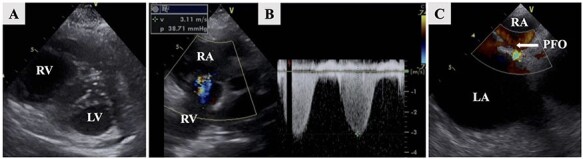
Transthoracic echocardiography shows left ventricular retraction due to right ventricular dilation (**A**). TR flow was 3.1 m/sec and the TR-PG was 38.7 mmHg (**B**). Transesophageal echocardiography revealed a PFO and shunt blood flow from the right-to-left atrium (**C**, arrow).

**Figure 2:**
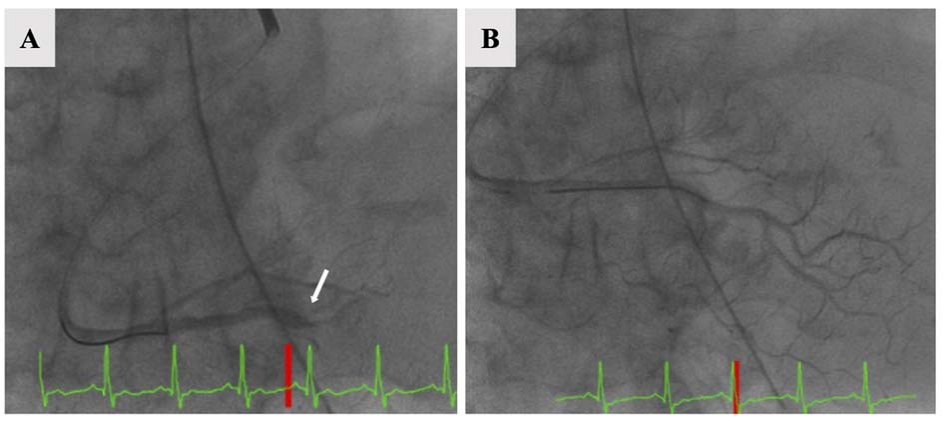
Coronary angiography revealed a 100% thrombus occlusion of 4PD (**A**, arrow) and no obvious stenosis of the other coronary arteries. Balloon dilatation and thrombus aspiration provided complete reperfusion with Grade 3 TIMI blood flow for 4PD occlusion without stenting (**B**).

**Figure 3:**
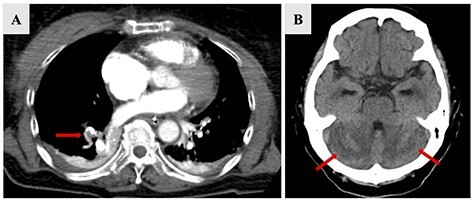
Chest CT with contrast revealed filling defects of the right peripheral pulmonary artery (**A**, arrow). The head CT on Day 5 revealed hypodensities in the bilateral cerebellar hemispheres (**B**, arrow).

A 77-year-old woman with a history of hypertension felt severe dyspnea after waking up in the morning and called emergency medical services. When the paramedics arrived, she had collapsed, and an electrocardiogram revealed asystole. Cardiopulmonary resuscitation was started, and she was transferred to the emergency department of our hospital. The return of spontaneous circulation was confirmed 60 minutes after her collapse. Her electrocardiogram showed ST elevation in leads II, III and aVF. Transthoracic echocardiography showed asynergy of left ventricle lower wall motion, left ventricular retraction due to right ventricle dilation (D-shape); tricuspid regurgitation (TR) flow was 3.1 m/sec, and the trans-tricuspid pressure gradient (TR-PG) was 38.7 mmHg (Fig. 1A and B). In addition, regional wall motion abnormality of the basal and mid right ventricular free wall with apical hyper contractility (McConnell’s sign) was observed. Based on these findings, he was suspected to have an AMI complicated by an APE. An emergency percutaneous coronary intervention (PCI) for AMI was performed. Coronary angiography revealed 100% thrombus occlusion of the distal segment of the right coronary artery (RCA, 4PD) and no obvious stenosis of the other coronary arteries. Balloon dilatation and thrombus aspiration provided complete reperfusion with Grade 3 TIMI blood flow for 4PD occlusion without stenting (Fig. 2). A subsequent whole-body computed tomography (CT) was performed to look for thrombosis and determine the cause of the pulmonary embolism. A contrasted chest CT revealed filling defects in the right peripheral pulmonary artery (Fig. 3A), and bilateral deep vein thrombosis was found in the lower limbs. The patient was admitted to the intensive care unit for post-resuscitation management. Initially, a right-to-left shunt involved in the mechanism of the concurrent AMI and APE was suspected. Transesophageal echocardiography revealed a PFO and blood flow from the right to the left atrium (Fig. 1C). Despite the absence of sedation and stable hemodynamics, she remained unconscious. The brain CT did not show an abnormality on admission; however, a follow-up brain CT revealed hypodensities in the bilateral cerebellar hemispheres on Day 4 (Fig. 3B). Based on these findings, we diagnosed APE, AMI and AIS. The suspected mechanism of these multiple embolisms was paradoxical embolism due to a PFO. We administered intravenous heparin for her multiple thrombi. Although there was possibility to be underlying some malignancy and PFO closure was a curative treatment, because of her severe neurological dysfunction caused by the ischemic stroke, no further tests were performed and her family did not consent to PFO closure. She left the intensive care unit on Day 9 and was discharged from the hospital on Day 53 with supportive care taking edoxaban 30 mg daily.

## DISCUSSION

We considered the following hypotheses regarding the development of multiple paradoxical embolisms in this case. Deep venous thrombus led to APE and caused pulmonary hypertension, which elevated right atrial pressure. The elevated right atrial pressure opened a PFO and caused a right-to-left shunt. The residual thrombus crossed the PFO from the right to the left atrium and entered the arterial circulation, causing an embolic myocardial infarction. Her sudden onset of dyspnea without chest pain and subsequent cardiopulmonary arrest with asystole suggested APE than typical AMI. The transthoracic and esophageal echocardiogram and the coronary angiography findings suggested these hypotheses. Subsequent AMI was explained by paradoxical embolism due to a PFO with elevated right atrial pressure. The AIS in bilateral cerebellar hemispheres was suggestive of a watershed infarct and may have been caused by global hypoperfusion, presumably at the time of cardiac arrest due to AMI and APE. It was reasonable to assume that these APE, AMI and AIS had developed in this sequence of events.

Anti-thrombotic therapy such as tissue plasminogen activator should be considered first treatment when patients present with concomitant AMI and APE. However, we could not administer anti-thrombotic drugs because she had a high risk of bleeding related to pulmonary contusion after chest compressions in this case. Therefore, we decided to perform PCI because usage of intravenous heparin for underlying the catheterization can be treated with pulmonary embolism simultaneously. In addition, if the patient’s condition deteriorates, extracorporeal membrane oxygenation can be used safely in the catheterization laboratory. The administration of anticoagulation therapy for venous thrombosis and PFO closure is secondary prevention for paradoxical embolisms [3]. Although theoretically it may help prevent further recurrence of paradoxical embolism, a PFO may serve to offset the elevated right atrial pressure and maintain cardiac output at the expense of low systemic saturation [8]. Little evidence exists to guide the decision to close a PFO in such a situation [9]. Prompt clinical diagnosis and targeted therapies adapted for the specific clinical presentation may have averted a fatal outcome.
